# Optimization of a direct spectrophotometric method to investigate the kinetics and inhibition of sialidases

**DOI:** 10.1186/1471-2091-13-19

**Published:** 2012-10-02

**Authors:** Jasvinder Kaur Hayre, Guogang Xu, Luisa Borgianni, Garry L Taylor, Peter W Andrew, Jean-Denis Docquier, Marco R Oggioni

**Affiliations:** 1 Dipartimento di Biotecnologie, Università degli Studi di Siena, I-53100, Siena, Italy; 2 Department of Respiratory Medicine, The Second Affiliated Hospital, Nanchang University, Nanchang, Jiangxi, 330006, China; 3 Department of Pulmonary Medicine, Beijing 301 Hospital, Beijing, 100853, China; 4 Biomedical Sciences Research Complex, University of St Andrews, St Andrews, KY16 9ST, UK; 5 Department of Infection, Immunity and Inflammation, University of Leicester, Leicester, LE1 9HN, UK

**Keywords:** Sialidase, Neuraminidase, Chromogenic sialic acids, Kinetic assay, Streptococcus pneumoniae

## Abstract

**Backgrounds:**

*Streptococcus pneumoniae* expresses three distinct sialidases, NanA, NanB, and NanC, that are believed to be key virulence factors and thus, potential important drug targets. We previously reported that the three enzymes release different products from sialosides, but could share a common catalytic mechanism before the final step of product formation. However, the kinetic investigations of the three sialidases have not been systematically done thus far, due to the lack of an easy and steady measurement of sialidase reaction rate.

**Results:**

In this work, we present further kinetic characterization of pneumococcal sialidases by using a direct spectrophotometric method with the chromogenic substrate *p*-nitrophenyl-N-acetylneuraminic acid (*p-*NP-Neu5Ac). Using our assay, the measured kinetic parameters of the three purified pneumococcal sialidase, NanA, NanB and NanC, were obtained and were in perfect agreement with the previously published data. The major advantage of this alternative method resides in the direct measurement of the released product, allowing to readily determine of initial reaction rates and record complete hydrolysis time courses.

**Conclusion:**

We developed an accurate, fast and sensitive spectrophotometric method to investigate the kinetics of sialidase-catalyzed reactions. This fast, sensitive, inexpensive and accurate method could benefit the study of the kinetics and inhibition of sialidases in general.

## Background

Sialidases catalyze the removal of terminal sialic acid residue from various glycoconjugates and have been implicated in pathogenesis of infectious diseases [1]. In fact, sialidases differ significantly in kinetic parameters, substrate specificity and catalytic properties. For example, typical sialidases hydrolyze sialiosides to release N-acetylneuraminic acid (Neu5Ac), whereas the leech intramolecular (IT) *trans*-sialidase produces 2,7-anhydro-Neu5Ac selectively from α2,3- sialosides, while trypanosomal *trans*-sialidase can also transfer Neu5Ac to another sugar [2,3].

The major human pathogen *Streptococcus pneumoniae* encodes three distinct sialidases, NanA, NanB and NanC that could be classified into three different subtypes [4]. According to a recent NMR report, NanA is a classic hydrolytic sialidase, whereas NanB could be an IT *trans*-sialidase similar to the leech enzyme, and NanC can handle the dual functions of both producing 2-deoxy-2,3-didehydro-N-acetylneuraminic acid (Neu5Ac2en, DANA) and hydrating this general sialidase inhibitor when substrate is depleted [5-7]. Nonetheless, it is proposed that the three could share a common catalytic mechanism before the final product formation step from a chemistry point of view. Based on these findings, a new sialidase triad is speculated, which might coordinate the sialidase action associated with pneumococcal virulence. However, the kinetic investigations of the three sialidases have not been systematically done thus far, due to the lack of an easy and steady measurement of sialidase reaction rate.

**Figure 1:**
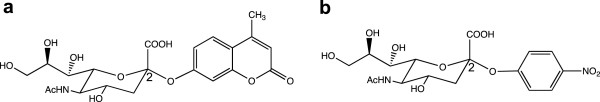
** The structures of 4MU-Neu5Ac and*p*NP-Neu5Ac.** (**a**) 4MU-Neu5Ac, Formula: C_21_H_24_NNaO_11_; MW: 489.41 g/mol; (**b**) *p*NP-Neu5Ac, Formula: C_17_H_22_N_2_O_11_; MW: 430.36 g/mol.

A variety of techniques have been used for the sialidase activity assays, but all present technical challenges. The classical method is the thiobarbituric acid (TBA) assay, in which released Neu5Ac reacts with TBA reagent and shows a specific absorbance measurable at 549 nm [8]. Thin layer chromatography (TLC) is another method to visualize the sialidase reaction products [9]. A more sophisticated method has been described by Trignali et al., which use radiolabelled gangliosides and high performance TLC separation of the reaction products [10]. Both methods are still employed for the sialidase substrate specificity determination. NMR is the best way to characterize the different sialic acids released, but requires access to expensive instrumentation [3,11]. In recent years, two artificial substrates 2’-(4-methylumbelliferyl)-α-D-N-acetylneuraminic acid (4MU-Neu5Ac) and 2-*O*-(*p*-nitrophenyl)-N-acetylneuraminic acid (*p-*NP-Neu5Ac) (Figure 1), were developed for fast sialidase assays [12]. The α-glycosidical linkage of these substrates can be hydrolyzed by sialidases to release a measurable fluorescence (4-methylumbelliferone, 4MU) or yellow product in alkaline conditions (p-nitrophenol, *p-*NP), respectively. 

To ease the measurement of reaction rates, and on the basis of the spectral properties of the reaction substrate (*p-*NP-Neu5Ac) and product (*p-*NP), a direct spectrophotometric method was designed in the current study, which allows the monitoring of the concentration of the reaction product as a function of time. In contrast to previous method, it does not need to stop the reaction by alkaline buffer before every reading. The kinetic characterization of NanA, NanB and NanC was performed, as it could provide further insights into their roles in pneumococcal virulence and metabolism [13-16]. The data are in good agreement with previously obtained NanA, NanB and NanC kinetic parameters, and followed the first-order reaction kinetics [5,6]. The anti-influenza drugs Zanamivir and Oseltamivir (inhibitors of influenza virus sialidases) were also tested as inhibitors of pneumococcal sialidases, in our experimental setup.

## Methods

### Cloning, expression and purification of *S. pneumoniae* sialidases

The genes *nanA* (spr1536) and *nanB* (spr1531) were cloned from the genomic DNA of *S. pneumoniae* strain R6. The third sialidase gene, *nanC*, which is not present in R6, so was obtained from TIGR4 pneumococcal DNA (SP1326). The amplified gene segments were subsequently ligated into the commercially-available vectors, PQE30 (Qiagen), PET23b (Novagen) and PET21b (Novagen), respectively. Recombinant proteins NanA, NanB and NanC were overexpressed in *Escherichia coli* systems and were purified as described previously [5,6,17]. Protein purity was monitored by SDS-PAGE and the protein identities were confirmed by mass spectrometry.

### Sialidase assays and determination of kinetic parameters

The activity of the sialidases was assayed colorimetrically and fluorometrically using the substrates *p-*NP-Neu5Ac (Sigma, St. Louis, Miss) and 4MU-Neu5Ac (Sigma, St- Louis, Miss), respectively. Conventional indirect assays were performed as previously described [5,6,17]. Briefly, the reaction mixtures containing the substrates and up to 100 nM sialidase were incubated at 37°C and stopped by the addition of 0.5 M Na_2_CO_3_ pH 9.0 for the colorimetric assay or 0.25 M glycine pH 10.0 for the fluorimetric assay. Released *p*-NP was detected spectrophometrically at 405 nm. The fluorescence associated with the release of 4-MU was measured at the excitation wavelength of 365 nm and an emission wavelength of 445 nm. These measurements were performed using an EnVision microplate reader (Perkin Elmer, Waltham, Mass.).

An alternative direct spectrophotometric method was optimized on the basis of the properties of the reaction substrate *p-*NP-Neu5Ac and its product *p-*NP, whose UV–vis spectra were recorded on a Cary 100 UV–vis spectrophotometer at 30°C. This method does not need to stop the reactions with the alkaline buffer and can be operated easily in multi-well flat-bottom assay plates and a microplate reader, allowing for a substantial improvement of data throughput. The variation occurring in the UV-visible spectrum upon hydrolysis of the substrate was investigated by recording, at different incubation times, spectra of 200–400 μM of *p-*NP-Neu5Ac in 10 mM MES buffer (pH, 6.0) in the presence of 25–700 nM of purified recombinant NanA.

The kinetic parameters of the various sialidases was determined at at 30°C in 20 mM MES buffer, pH 6.0. The initial reaction rates or complete time-course hydrolyses were recorded at a fixed wavelength of 400 nm (ΔɛM, 1,300 ± 200 M^-^1·cm^-1^) in the presence of various substrate concentrations. Enzyme concentrations were adjusted to obtain a measurable initial velocity or to record the complete reaction time-course (E_0_ ranged 14 to 140 nM for NanA; 160 to 9,600 nM for NanB; and 100 to 500nM for NanC). Kinetic parameters were computed using either the direct fit of the initial rates *vs* [S] data with the Henri-Michaelis-Menten equation of by analyzing the complete time-course reactions with the integrated form of Henri-Michaelis-Menten equation, as previously described [18]. Each reaction was repeated at least three times. The pH and buffer dependencies of the sialidases NanA, NanB NanC were investigated in the buffers (Sodium Citrate/Disodim phosphate (100 mM, pH 4.0-5.5) and MES [2-(*N*-morpholino) ethanesulfonic acid] (20 mM, pH 5.5-7.0). Sialidase inhibitors used in this study, Neu5Ac2en, Zanamivir and Oseltamivir carboxylate were obtained from Sigma, GlaxoSmithKline and Roche Pharmaceutics respectively. Inhibition constants (*K*_*i*_s) were computed by measuring the initial reaction rates in the presence of varying concentration of inhibitors, using an competitive inhibition model, as previously described (20). Alternatively, the V_max_/*K*_m_* value, where *K*_m_* = *K*_*m*_(1 + [I]/*K*_*i*_), was computed from the complete time-course hydrolysis in the presence of various inhibitor concentrations. The ratio (V_max_/*K*_*m*_)/( V_max_/*K*_m_*) (in the i. e. in the absence and presence of the inhibitor, respectively) was plotted as a function of [I], yielding a line, whose slope corresponds to 1/*K*_*i*_.

### Thermal stability assay of sialidase NanA

The thermal shift assay of sialidase NanA against different buffers was performed in an iCycler iQ5 Real-Time PCR Detection System (Bio-Rad, Hercules, CA). In brief, to each well of a 96-well PCR plate was added 1 μl of 50 μM NanA, 0.25 μl of 500 × Sypro orange protein dye (originally 5000 × in Dimethyl sulfoxide) and 48.75 μl of buffer. The final volume was 50 μl. After sealing with optical film on top, the plate was put into the Real-Time PCR machine. The plate was then heated from 25°C to 89°C at the heating rate of 0.5°C per min. The fluorescence intensity of each well was measured at 490 nm excitation and 530 nm emission wavelength every min. The thermal shift curves were generated by the iCycler system automatically.

## Results

### Optimization of a direct spectrophotometric assay to investigate the kinetics of sialidase-catalyzed reactions

**Figure 2:**
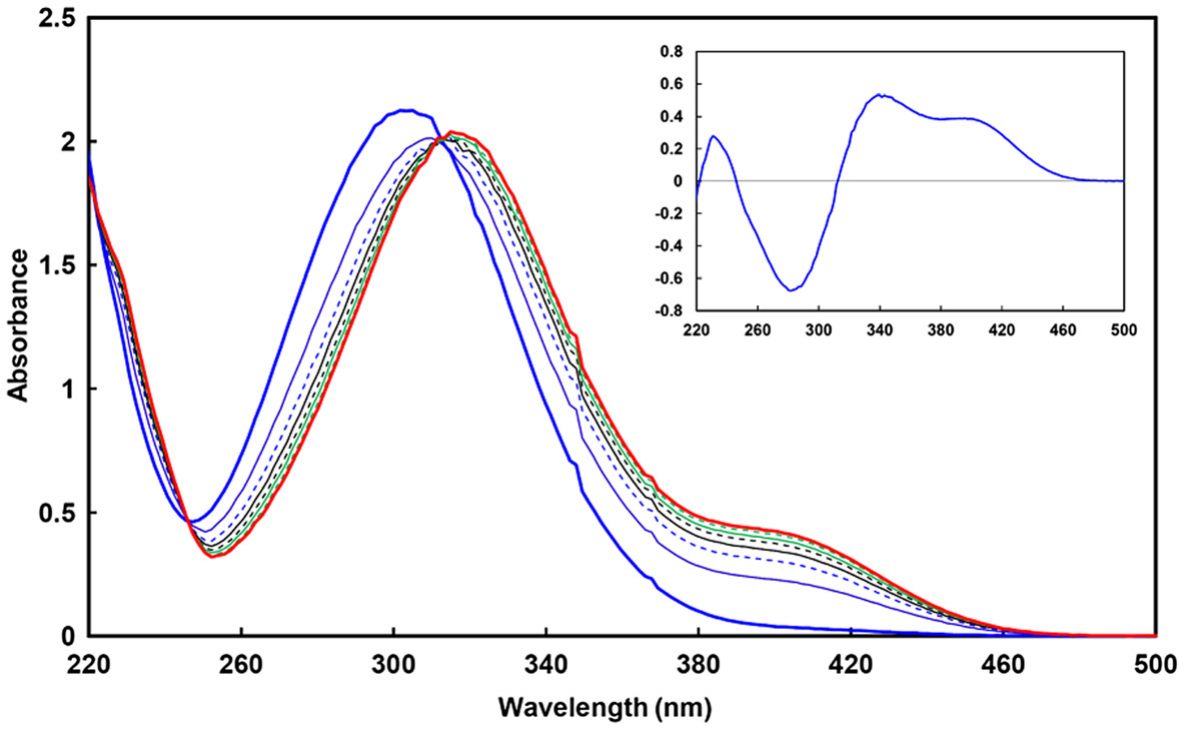
** UV–vis spectra of*p-*NP-Neu5Ac upon hydrolysis by the purified sialidase NanA.** Spectra were recorded for 232 μM pNP-Neu5Ac alone (thick blue line) and after 1 (thin blue line), 2 (dashed blue line), 3 (black line), 4 (dashed black line), 5 (green line), 7 (dashed green line) and 15 min (thick red line) incubation in the presence of 100 nM NanA. The reaction was complete after 15 min and the spectra did not change upon further incubation. **Inset.** Difference spectrum between intact and hydrolyzed *p-*NP-Neu5Ac, showing the variation in the spectrum: the appearance of a signal at 400 nm and a red-shift of the main peak from ~300 to ~315 nm.

**Figure 3:**
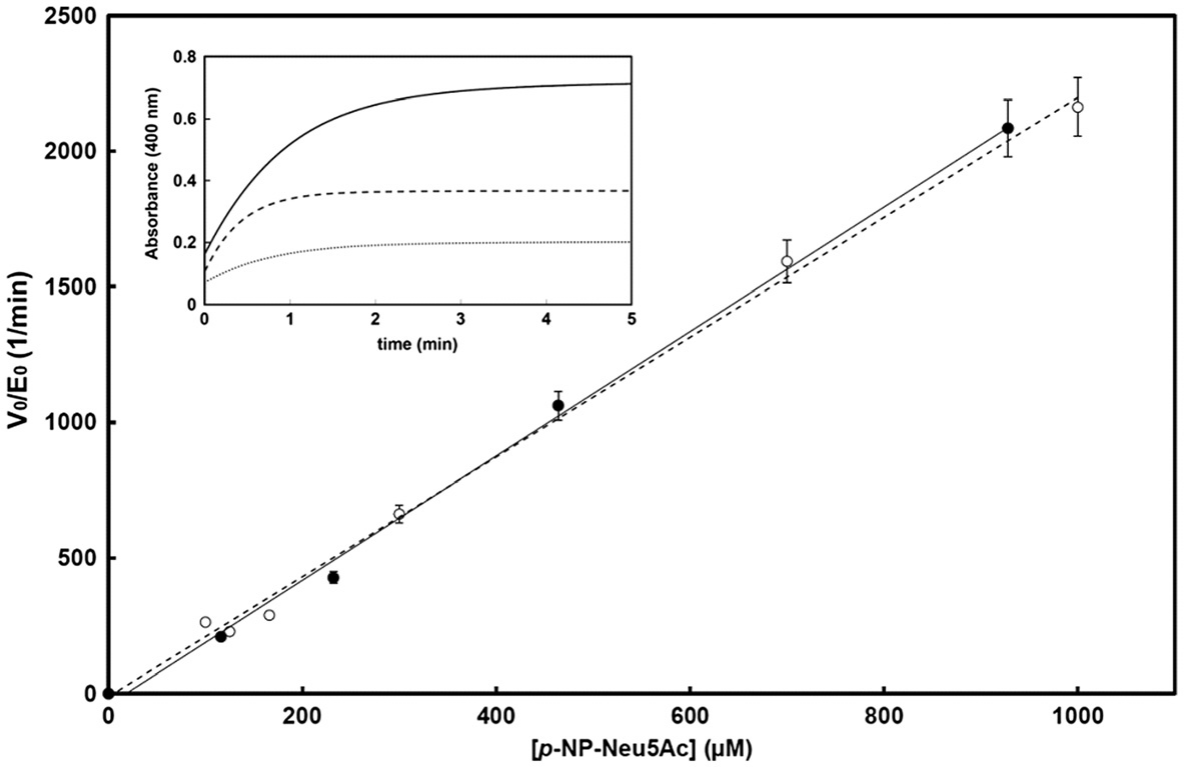
** Henri-Michaelis-Menten plot for the hydrolysis of*p-*NP-Neu5Ac by NanA.** The normalized initial rates (V_0_/E_0_) are plotted as a function of substrate concentration, showing the perfect agreement between the initial rates measured with the conventional indirect method (empty circles) and our direct method (black circles). Reactions followed a first-order kinetics. **Inset**. An example of complete hydrolysis time course of 116, 232 and 464 μM *p-*NP-Neu5Ac (dotted, dashed and plain line, respectively) by 100 nM NanA, which allowed to compute the catalytic efficiency as described by DeMeester *et al.*[18]

UV-visible spectra of the substrate and reaction product of pneumococcal sialidases catalysis were compared in this work. The production of *p*-NP resulted in the appearance of an absorbance signal at 400 nm, an increase of absorbance at 220 nm and a red-shift of the substrate peak at ~300 nm to ~315 nm (Figure 2). The difference spectrum showed that major absorbance variations upon substrate hydrolysis were observed at 350 and 400 nm.To readily measure initial reaction rates and record complete hydrolysis curves, a fixed wavelength of 400 nm was chosen as (a) the substrate does not significantly absorb at this wavelength and (b) it is in the visible range. The molar variation of the extinction coefficient was computed from the difference spectrum (ΔεM, 1,300 ± 200 M^-^1·cm^-1^). . Although the absorbance variation is lower than with the indirect spectrophotometric method (the extinction coefficient of p-NP at pH 9.0 is ~18,000 M^-1^·s^-1^), the final data were in good agreement with previous NanA, NanB and NanC kinetic results. The kinetic parameters were computed using the direct fit of the data (initial reaction rates *vs* substrate concentration) with the Henri-Michaelis-Menten equation. As shown Figure 3 and Table 1, it can be seen that all reactions clearly followed first-order kinetics and allowed to compute the *k*cat/*Km* parameter, but not the *k*_cat_ and *K*_m_ values. These kinetic parameters with the direct method are comparable to those obtained with the conventional assays carried out previously with the indirect method [16] (Figure 2, Table 1) and also with the previously published NMR kinetic results using α2,3-sialyllactose as a substrate [6]. Furthermore, the three pneumococcal sialidases present distinct activity: NanA is most active sialidase among the three, the catalytic efficiency of which is at least 10 times higher than NanB and NanC in this assay. 

**Table 1:** Kinetic parameters for the hydrolysis of p-NP-Neu5Ac by the pneumococcal sialidases computed using the direct and the indirect spectrophotometric assays (see Materials and Methods for details)

	***p-*** **NP-Neu5Ac direct method**			**p-NP-Neu5Ac indirect method**		
	***k***_***cat***_**(s**^**-1**^**)**	***K***_***m***_**(μM)**	***k***_**cat**_**/*K***_***m***_**(M**^**-**^**1·s**^**-1**^**)**	***k***_***cat***_**(s**^**-1**^**)**	***K***_***m***_**(μM)**	***k***_**cat**_**/*K***_***m***_**(M**^**-**^**1·s**^**-1**^**)**
NanA*	>175	>500	(3.5 ± 0.3) × 10^5^	>490	>1,100	(8.4 ± 0.9) × 10^4^
NanB*	>0.14	>500	(2.7 ± 0.3) × 10^2^	>11	>1,200	(1.1 ± 0.1) × 10^2^
NanC*	>17	>500	(3.4 ± 0.3) × 10^4^	>11	>700	(1.2 ± 0.1) × 10^4^

* first order reaction kinetics were observed with this substrate at concentrations up to 500 μM (direct method) or 1,200 μM (indirect method) and thus only allowed to compute the *k*_cat_/*K*_*m*_ values.

### The effects of buffer and pH on the activity of pneumococcal sialidases

**Figure 4:**
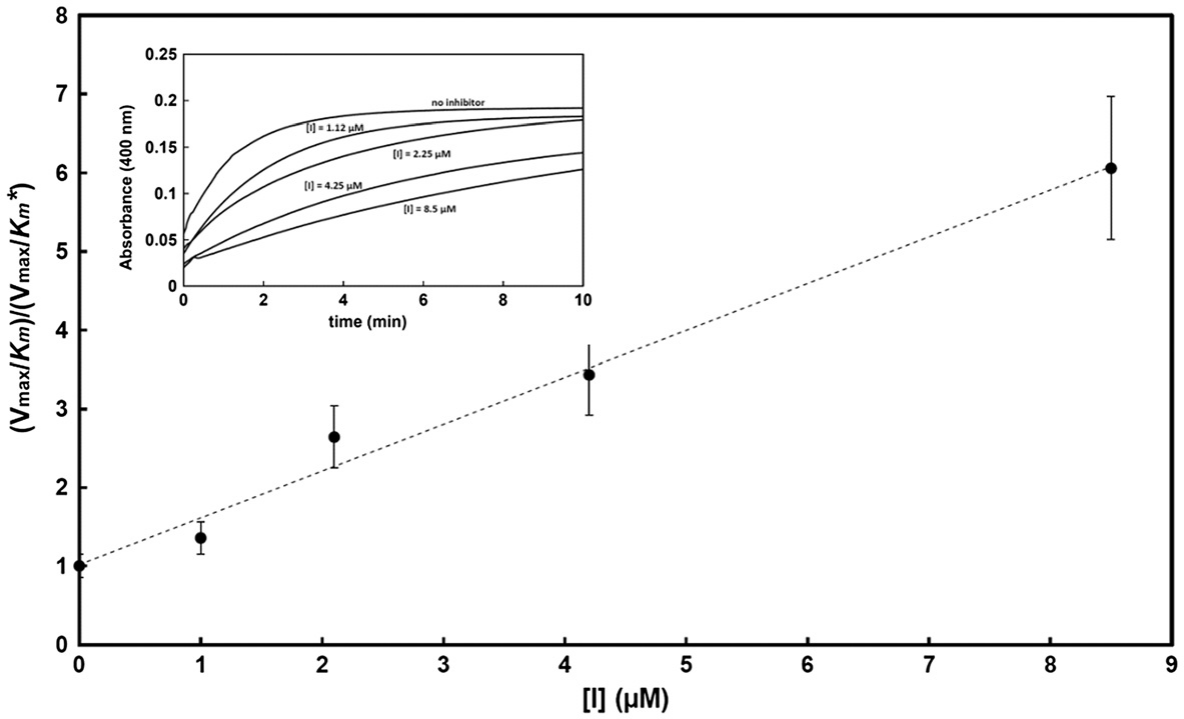
** Inhibition of NanA by the sialidase inhibitor Neu5Ac2en.** The graph shows the dependence of the (V_max_/*K*_*m*_)/(V_max_/*K*_m_*) ratio as a function of the inhibitor concentration. The V_max_/Km* values were computed from the complete time-course reactions recorded in the presence of increasing concentrations of inhibitor and using 232 μM *p-*NP-Neu5Ac as the reporter substrate.

**Figure 5:**
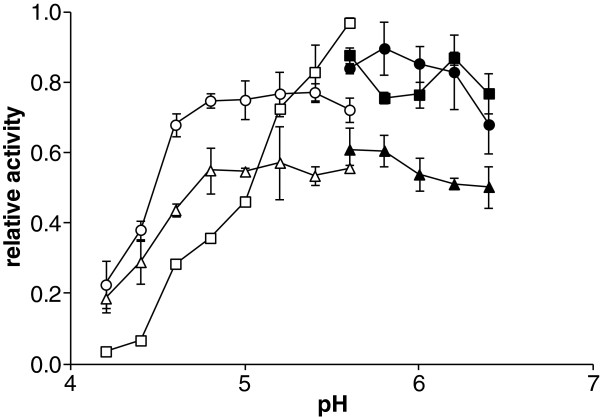
** The buffer/pH effects on the pneumococcal sialidase activity.** The three sialidases NanA (squares), NanB (circles) and NanC (triangles) showed a variable activity in different buffers (empty markers, citrate/phosphate buffer; plain markers, MES buffer) and at pH values ranging from 4–6.5, with optimum pHs which varied slightly among the three enzymes (NanA 5.5-6.5; NanB 5–5.5; NanC 5–6). These data, measured with either the fluorogenic substrate 4MU-Neu5Ac or the chromogenic substrate *p*-NP-Neu5Ac, gave comparable results.

**Figure 6:**
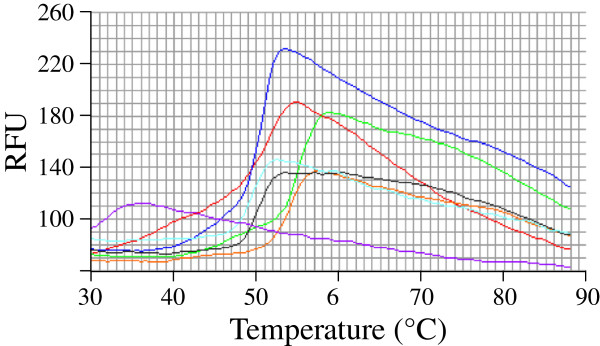
** The thermal shift assay of NanA in seven different buffers.** The melting curves of NanA undergo unfolding with the increasing of temperature in the tested buffers (Red Acetate, Orange MOPS, Blue Phosphate, Green MES, Cyan HEPES, Black Tris-Cl, and Purple Imidazole). MES buffer (Green line) confers the optimal sialidase thermal stability for NanA.

Previously we identified that CHES (2-n-cyclohexylamino ethane sulfonic acid) buffer is a weak inhibitor of pneumococcal sialidases [5]. In this study, to avoid using buffers that might affect the enzyme activity, a kinetic study with some commonly used buffer systems, such as citric phosphate and MES, and was performed. It was observed that all the sialidases are active in a wide pH range from 4–8, but the pH optimum varied slightly among the three enzymes (NanA 5.5-6.5; NanB 5–5.5; NanC 5–6, see Figure 4). This finding is also in agreement with the preliminary thermal shift assay screening results, which showed NanA present higher thermal stability in MES than in other buffer systems (Figures 5 and 6). 

### Inhibition of pneumococcal sialidases by three common neuraminidase inhibitors

With our modified method, the inhibition of NanA, NanB and NanC by the general sialidase inhibitor Neu5Ac2en and the influenza virus sialidase inhibitors (Zanamivir and Oseltamivir carboxylate) was readily investigated. Only Neu5Ac2en and Oseltamivir carboxylate showed significant inhibition of NanA, with inhibition constants in the micromolar range (Figure 4, Table 2). Very limited inhibitory effects of NanA were seen with Zanamivir. NanB and NanC appeared to be poorly or not inhibited by the three sialidase inhibitors. These findings are in good agreement with previous reports [19]. 

**Table 2:** Inhibition assay of pneumococcal sialidases

	***K***_***i***_**(μM)**		
	**Neu5Ac2en**	**Oseltamivir carboxylate**	**Zanamivir**
NanA	1.6	1.77	720
NanB	330	-*	-*
NanC	2,010	-*	-*

*-, no inhibition observed with inhibitor concentrations up to 7,500 μM.

## Discussion

Sialidases play a key role in infections caused by several microbial pathogens including influenza virus, parainfluenza virus and *S. pneumoniae*[1]. Therefore, a reliable, fast, simple, inexpensive and accurate kinectic method would be of advantage for characterizing the potential inhibitors or pro-drugs for sialidases, a major target for drug redevelopment [1]. Indeed, although the classical TBA and TLC are useful in providing key information regarding substrate specificity, these assays have major drawbacks represented by the complicated experimental procedures and the use of highly toxic reagents, such as arsenite (Table 3) [8,9]. Other methods, such as NMR spectroscopy, would be perfect to detect small-molecule interactions and determine the final product, but needs a high-profile NMR facility and large amount of substrates and enzymes, as well as well-trained NMR personnel [11,20]. Therefore, most researchers preferred using the artificial substrates 4MU-Neu5Ac or *p-*NP-Neu5Ac for sialidase enzyme kinetics. When the α-glycosidical linkage is hydrolyzed by sialidases, the reactions will be stopped by adding some extreme pH buffer and the leaving groups 4MU or *p-*NP can be quantified by fluorimetry or spectrophotometry respectively. 4MU-Neu5Ac is relatively expensive and is prone to degradation at room temperature. Furthermore, the presence of Neu5Ac2en can interfere with the fluorescence reading of 4MU. Therefore, we focused on the modification of the *p-*NP-Nue5Ac method to establish a better approach for sialidase enzyme kinetics. 

**Table 3:** Comparisons of existing sialidase assay methods

	**TBA assay**	**TLC**	**Radiochemical method**	^**1**^**H-NMR**	**HPLC**	**Fluorescence assay**	**Spectophotometric assay**
**Substrates**	Sialosides	Sialosides	Sialosides	Sialosides	Sialosides	4MU-Neu5Ac	*p*NP-Neu5Ac
**Reagents**	Arsenite, TBA	Resorcinol or diphenylamine	Radiolabelled gangliosides	Deuterated buffer	Malonitrile	Normal buffer	Normal buffer
**Instruments**	Spectrometer	TLC plate	HPTLC and radiochromatoscanner	NMR facility	HPLC and fluorimeter	Fluorimeter	Spectrophotometer
**Time to run**	hours	hours	hours	hours	hours	10 min	10 min
**Safety issue**	5TC toxic	Hazardous in case of skin contact	Radioactive substrate	Strong magnet	None	None	None
**Automation**	No	No	No	No	Yes	Yes	Yes
**Cost per run**	Cheap	Cheap	Expensive	Very Expensive	Expensive	Expensive and substrate unstable	Cheap
**Substrate specificity**	Yes	Yes	Yes	Yes	Yes	No	No
**Enzyme kinetics**	Inaccurate; Time consuming	Not applicable	Costly; Time consuming	Costly; Time consuming	Costly; Time consuming	Yes	Yes
**Reference(s)**	[8]	[9]	[10]	[3,11]	[20]	[7,12,16]	[12]

Here, with both 4MU-Neu5Ac and *p-*NP-Neu5Ac sialidase assays and our direct spectrophotometric method, we observed that NanA, NanB and NanC show different pH optima and the activity could be affected by the buffer systems. It is intriguing that phosphate buffer at neutral pH has some negative effects on the NanA and NanC activity, but not NanB. By contrast, the three sialidases shower higher enzymatic activity in MES buffer, pH 5.5. Preliminary thermal shift assays confirmed that MES could increase the thermal stability of the sialidase.

In the present study, we also investigated the typical sialidase inhibitors Neu5Ac2en, Oseltamivir carboxylate and Zanamivir. Unlike the influenza virus sialidases, NanA is the only enzyme among the three pneumococcal sialidases that could be inhibited by Neu5Ac2en and Oseltamivir carboxylate with inhibition constants in the micromolar range [19]. Very limited inhibitory effects of NanA were seen with Zanamivir. None of the tested inhibitors significantly inhibited NanB and NanC (*K*_*i*_ > > 7.5 mM). Previous structural studies had indicated that the guanidinium group of Zanamivir does not add to substrate-NanA interactions, which is different from the binding pattern of influenza virus neuraminidase and could be the reason why Zanamivir is a weak inhibitor to NanA [19]. Similar results were also observed in animal studies [14]. Furthermore, comparison of NanA complexes with NanB structures and NanC model shows that both Zanamivir and Oseltamivir cannot interact tightly with the other pneumococcal sialidases due to the different architecture around active sites [19].

## Conclusions

In this work, we developed an accurate, fast and sensitive spectrophotometric method to investigate the kinetics of sialidase-catalyzed reactions. Using our assay, the measured kinetic parameters of the three purified pneumococcal sialidase, NanA, NanB and NanC, were obtained and were in perfect agreement with the previously published data [5-7]. In view of the fact that both the substrates used in this paper, as also the relative inhibitors work well, and were in fact originally developed, for assays of viral sialidases, the method developed should be suitable for the assays of most sialidases, including most if not all viral and bacterial sialidases [21,22]. This method, prone to automation and high-throughput screening, could ease and accelerate the screening of potential large libraries of chemical compounds to identify new inhibitors, which represent interesting and relevant anti-infective drug targets.

## Abbreviations

*p-*NP-Neu5Ac: *p*-nitrophenyl-N-acetylneuraminic acid; Neu5Ac: N-acetylneuraminic acid; Neu5Ac2en: 2-deoxy-2,3-didehydro-N-acetyl- neuraminic acid; 4MU-Neu5Ac: 2’-(4-methylumbelliferyl)-α-D-N-acetylneuraminic acid.

## Authors’ contributions

JKA, GX, LB and JDD performed the experiments. JDD, GLT and PWA participated in the study design. JDD and MRO supervised the work and defined the study design. GX, JDD and MRO wrote the manuscript. All authors read and approved the final manuscript.
